# Alterations in Vaginal Microbiota and Associated Metabolome in Women with Recurrent Implantation Failure

**DOI:** 10.1128/mBio.03242-19

**Published:** 2020-06-02

**Authors:** Min Fu, Xiaowei Zhang, Yiheng Liang, Shouren Lin, Weiping Qian, Shangrong Fan

**Affiliations:** a Department of Obstetrics and Gynecology, Peking University Shenzhen Hospital, Shenzhen, Guangdong, China; b Department of Reproductive Medicine, Peking University Shenzhen Hospital, Shenzhen, Guangdong, China; c BGI-Shenzhen, Shenzhen, Guangdong, China; d Shenzhen Key Laboratory on Technology for Early Diagnosis of Major Gynecological Disease, Shenzhen, Guangdong, China; LA State University Health Sciences Center; University of Georgia

**Keywords:** recurrent implantation failure, RIF, vaginal microbiota, metabolome, *in vitro* fertilization, IVF, infertility

## Abstract

Recurrent implantation failure (RIF) refers to repeated failure to become pregnant after transferring embryos with normal morphology. However, the pathogenesis of RIF remains unrevealed, especially for those without any pathological features. In this study, we characterized the vaginal microbiota and metabolomes of patients with unexplained RIF, while patients who achieved clinical pregnancy in the first frozen embryo transfer (FET) cycle were used as controls. Based on 16S rRNA gene sequencing of the vaginal microbiota, the vaginal *Lactobacillus* showed a significant positive correlation with the pregnancy rate, and the RIF group presented higher microbial α-diversity than the control group (*P* value = 0.016). The metabolomic profile identified 2,507 metabolites, of which 37 were significantly different between the two groups (*P* value < 0.05, variable importance for the projection [VIP] > 1). Among them, 2′,3-cyclic UMP and inositol phosphate were the top two metabolites that were higher in the RIF group, while glycerophospholipids and benzopyran were important metabolites that were lower in the RIF group. A lack of lysobisphosphatidic acid and prostaglandin metabolized from glycerophospholipids will lead to deferred implantation and embryo crowding. Benzopyran, as a selective estrogen receptor modulator, may affect the outcome of pregnancy. All of the changes in metabolite profiles may result in or from the differential microbiota compositions in RIF patients. In conclusion, significant differences were presented in the vaginal microbiota and metabolomes between patients with unexplained RIF and women who became pregnant in the first FET cycle. For the first time, this study elaborates the possible pathogenesis of RIF by investigating the vaginal microbiota and metabolites in RIF patients.

## Abstract (precis)

*In vitro* fertilization-embryo transfer (IVF-ET) is now widely applied for treating infertility, and unexplained recurrent implantation failure (RIF) has become a substantial challenge. We hypothesize that vaginal microbial dysbiosis is associated with RIF, as it is linked to many female reproductive diseases. In this study, we characterized the vaginal microbiota and metabolomes of patients with unexplained RIF, while patients who achieved clinical pregnancy in the first IVF cycle were set as controls. In general, significant differences were discovered in the vaginal microbiota and metabolomes between the two groups. This study is the first detailed elaboration of the vaginal microbiota and metabolites associated with RIF. We believe that our findings will inspire researchers to consider the dynamics of microbiomes related to the microenvironment as a critical feature for future studies of nosogenesis not only for RIF but also for other reproductive diseases.

## INTRODUCTION

Infertility is defined as the inability to become pregnant by a couple who have normal intercourse without contraception for 1 year (1, 2). The incidence of infertility ranges from 8% to 12% in reproductive-aged couples, which has become a definite global public health issue (3). *In vitro* fertilization-embryo transfer (IVF-ET) is currently widely used in the treatment of infertility. In 1978, the first IVF baby was born in the United Kingdom. Over the intervening years, the success rate has steadily increased, with a cumulative live birth rate of >52% after 3 cycles of treatment (4). Nevertheless, IVF treatment failure still occurs frequently. Among all repeated IVF failure types, recurrent implantation failure (RIF) is a special subgroup. RIF refers to the women under the age of 40 years who received at least four good-quality embryos in a minimum of three fresh or frozen cycles that still failed to achieve a clinical pregnancy (5). Failed IVF usually occurs in elderly patients and those with a low ovarian reserve or low ovarian response. For them, high-quality embryos will be selected and transferred (5). However, many patients still fail to become pregnant after repeated IVF cycles. RIF has become a new and substantial challenge, as its occurrence rate among infertility patients has continued to increase in recent years (6). RIF also carries a heavy financial burden and deeply impacts the patient’s body and mind.

The investigation and management of RIF usually focuses on the quality of the embryo and endometrial receptivity. Recently, the importance of maternal systemic diseases, such as thyroid, thrombophilia, and immunological disorders, has also been recognized (7–9). Although many RIF patients have undergone plenty of clinical examinations and tried various possible treatments, they are still unable to become pregnant. Moreover, a recent paper reported that nonsenile patients still have a low live-birth rate (36.6%), even when euploid blastocyst transfer cycles are selected by comprehensive chromosome screening (CCS) and after confirming the window of implantation (WOI) by endometrial receptive array (ERA) (10). Even worse, another group of patients with unexplained RIF have no pathological features, for whom the treatment method is more intractable. Hence, the nosogenesis of RIF is complex and requires a multidimensional explanation.

The human commensal microbiome, referred to as “the other human genome,” coexisted and evolved with the human genome to help maintain human health. Microbial dysbiosis and invasion of pathogens can lead to disease and even threaten human life. Metabolic syndrome, diabetes (11, 12), obesity (13, 14), alcoholic liver disease (15), cirrhosis (16), coronary heart disease (17), and some mental diseases (18, 19) have been found to be related to intestinal microbial metabolism. With the development of high-throughput sequencing, the microbiome has also been characterized in the vaginal environment, even in the female upper reproductive tract, which has traditionally been considered sterile (20). Ravel et al. divided the vaginal flora into five community state types (CSTs) by 16S rRNA gene sequencing in 2011, four of which were classified as *Lactobacillus*-dominated (LD) types (21). *Lactobacillus* is one of the dominant genera in the vaginal microbiota of healthy females, and its key metabolites, which include lactate acid, can maintain the acidic and anaerobic vaginal environment and protect it from pathogen infection. A series of pregnancy-related diseases, such as premature rupture of membranes, premature delivery, and chorioamnion infection (22–26), diseases associated with infertility, such as diabetes, obesity, pelvic inflammatory disease (27), and sexually transmitted diseases (28), and even cervical cancer (29) have been discovered to be associated with vagina dysbiosis.

A previous systematic review reported that an abnormal vaginal microbiota is associated with tubal factor infertility and early spontaneous abortion in IVF patients. However, that article also pointed out that the quality of evidence was low and needs further research (30). In addition, because microbial compositions change along the female reproductive tract and are associated with pregnancy-related diseases, sexually transmitted diseases, infertility-related metabolic diseases, gynecological tumors, and so on (28, 31–33), we assume that the microbiota and its metabolites might be associated with every step of IVF, including gamete formation, implantation, and delivery (34, 35). In this study, we explored the association of microbial composition and function with IVF/RIF, and microbiomic and metabolomic analyses were both used as advanced tools. Metabolomics is the untargeted identification and quantification of all low-molecular-weight end products of cellular biological processes (36). The levels of metabolites ultimately reflect the integrated response of a biological system and directly influence the host. Additionally, individuals who achieved a successful pregnancy in the first frozen embryo transfer (FET) cycle were used as controls to explore the relationship of the microbial community and metabolites with RIF.

## RESULTS

### Diversity and composition of the vaginal microbiota.

A total of 67 samples were analyzed by 16S rRNA gene sequencing to investigate the vaginal microbiota, including 27 samples from the RIF group and 40 from the control group. The clinical information of the subjects is shown in Table 1. In total, 2,824,185 reads were obtained from these 67 samples, and on average, 42,152 ± 10,415 reads per sample and 424 ± 7 bp per read were achieved. After clustering, the rarefaction curve of the operational taxonomic unit (OTU) number was almost a straight horizontal line, which demonstrated that the samples were sequenced with enough depth in this study (see Fig. S1 in the supplemental material).

**TABLE 1:** Clinical characteristics of the participants in the two groups whose samples were submitted for 16S rRNA gene sequencing of the vaginal microbiota

Clinical characteristics	Value		*P* value
	RIF group (*n* = 27)	Control group (*n* = 40)	
Age (yrs)	33.4 ± 3.7	32.0 ± 4.0	0.213
BMI (kg/m^2^)	20.9 ± 3.4	22.3 ± 7.6	0.102
AMH (ng/ml)	3.0 ± 1.9	4.4 ± 3.8	0.101
Duration of infertility (yrs)	4.5 ± 3.0	3.7 ± 2. 7	0.068
Endometrial thickness (mm)	11.5 ± 1.8	12.1 ± 2.2	0.162
No. of oocytes	12.7 ± 5.9	12.2 ± 5.5	0.734
No. of embryos	9.7 ± 5.0	9.7 ± 4.4	0.847
No. of high-quality embryos	2.9 ± 2.1	2.8 ± 2.5	0.568
No. of embryos transferred	1. 9 ± 0.4	2.1 ± 0.4	0.044
No. of high-quality embryos transferred	1.3 ± 0.5	1.3 ± 0.9	0.576

10.1128/mBio.03242-19.1 FIG S1 Rarefaction curves of the sequenced samples. Download FIG S1, PDF file, 1.7 MB. Copyright © 2020 Fu et al. 2020 Fu et al. This content is distributed under the terms of the Creative Commons Attribution 4.0 International license.

**FIG 1:**
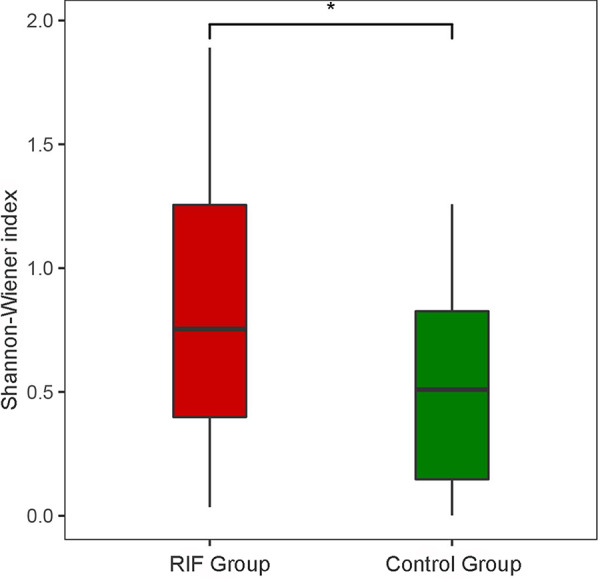
The α-diversity of the vaginal microbiota in the two groups was calculated and is shown by the Shannon-Wiener index.

A total of 804 OTUs were obtained in the two groups. The RIF group contained 730, and the control group contained 429, among which 355 were shared between the two groups. The numbers of OTUs found in each sample of the RIF group and control group were 71.48 ± 37.02 and 42.53 ± 14.51, respectively. The number of OTUs in the RIF group was much larger than that in the control group, indicating that the microbial composition of the RIF group was richer. The α-diversity of the microbiota was calculated by the Shannon-Wiener index (0.80 ± 0.50 for the RIF group and 0.50 ± 0.39 for the control group, *P* value < 0.01). Although some individuals in both groups showed similar levels of diversity due to the overlap of the Shannon-Wiener index values, the statistical analysis results still demonstrated that the microbial diversity in the vaginal environment was significantly higher in the RIF patients than in the control individuals (Fig. 1).

**FIG 2:**
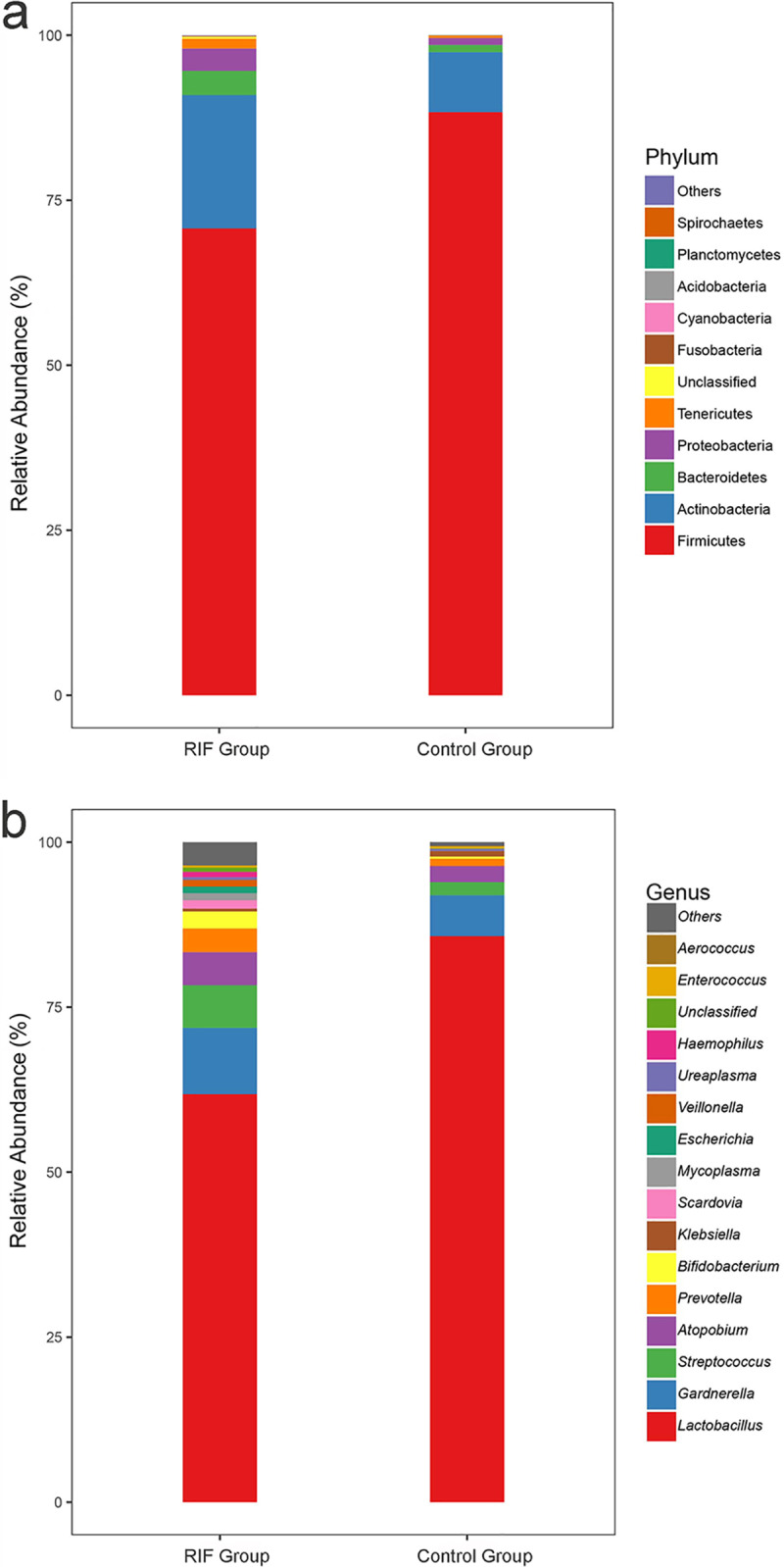
Taxonomic classification of the vaginal microbiota at the phylum level (a) and genus level (b) from the RIF and control groups.

The taxonomic classification at the phylum level showed similar patterns in the two groups, which were both dominated by *Firmicutes*, *Actinobacteria*, *Bacteroidetes*, *Proteobacteria*, and *Tenericutes* (*Mollicutes*); however, the relative abundances of *Firmicutes* and *Bacteroidetes* were significantly different (*P* value < 0.05) (Fig. 2a). At the genus level, genera with the top 15 abundances are shown in Fig. 2b. Among them, the abundances of *Lactobacillus*, *Gardnerella*, *Atopobium*, *Streptococcus*, and *Prevotella* were higher than 1% in both the control and RIF groups, while those of *Bifidobacterium*, *Scardovia*, *Mycoplasma*, and *Escherichia* were >1% only in the RIF group. There were 26 genera that were significantly different between the two groups, of which 5 were aerobes, 12 were anaerobes, and 9 were unclassified (see Table S1). *Lactobacillus*, as a main dominant genus, was also dissected in this study, and 12 species were discovered in the samples. The results showed that Lactobacillus crispatus and Lactobacillus iners were the most abundant *Lactobacillus* species in the RIF and control groups, respectively. The abundance of *L. iners* was significantly lower in the RIF group than in the control group (*P* value < 0.05) (see Table S2). However, the other 11 *Lactobacillus* species showed no significant differences.

10.1128/mBio.03242-19.4 TABLE S1 Genera in the vaginal microbiota that were significantly differentially abundant between the RIF and control groups. Download Table S1, DOCX file, 0.1 MB. Copyright © 2020 Fu et al. 2020 Fu et al. This content is distributed under the terms of the Creative Commons Attribution 4.0 International license.

10.1128/mBio.03242-19.5 TABLE S2 Relative abundance of *Lactobacillus* species discovered in the samples that were significantly different between the RIF and control groups. Download Table S2, DOCX file, 0.1 MB. Copyright © 2020 Fu et al. 2020 Fu et al. This content is distributed under the terms of the Creative Commons Attribution 4.0 International license.

The potential biomarkers for the predictive model of RIF occurrence were examined by the random forest algorithm among 18 genera with an abundance >0 in both groups out of 26 significantly different genera. Two targeted genera selected by the Gini coefficient, *Lactobacillus* and *Veillonella*, were sent for receiver operating characteristic (ROC) analysis (see Table S3). The identified *Lactobacillus* and *Veillonella* in patients with a diagnosis of RIF had a sensitivity of 0.627 and 0.560 and specificity of 0.775 and 0.900, respectively (see Fig. S2). Notably, the sensitivity and specificity both improved when the two genera were factored in (sensitivity = 0.633; specificity = 0.925).

10.1128/mBio.03242-19.2 FIG S2 ROC curve for *Lactobacillus* and *Veillonella* presence in the vagina. Download FIG S2, PDF file, 0.1 MB. Copyright © 2020 Fu et al. 2020 Fu et al. This content is distributed under the terms of the Creative Commons Attribution 4.0 International license.

10.1128/mBio.03242-19.6 TABLE S3 Gini coefficients calculated by a random forest algorithm among 18 genera with an abundance of >0 in two groups out of 26 significantly different genera. Download Table S3, DOCX file, 0.1 MB. Copyright © 2020 Fu et al. 2020 Fu et al. This content is distributed under the terms of the Creative Commons Attribution 4.0 International license.

### Distribution of the vaginal microbiota in all the samples.

**FIG 3:**
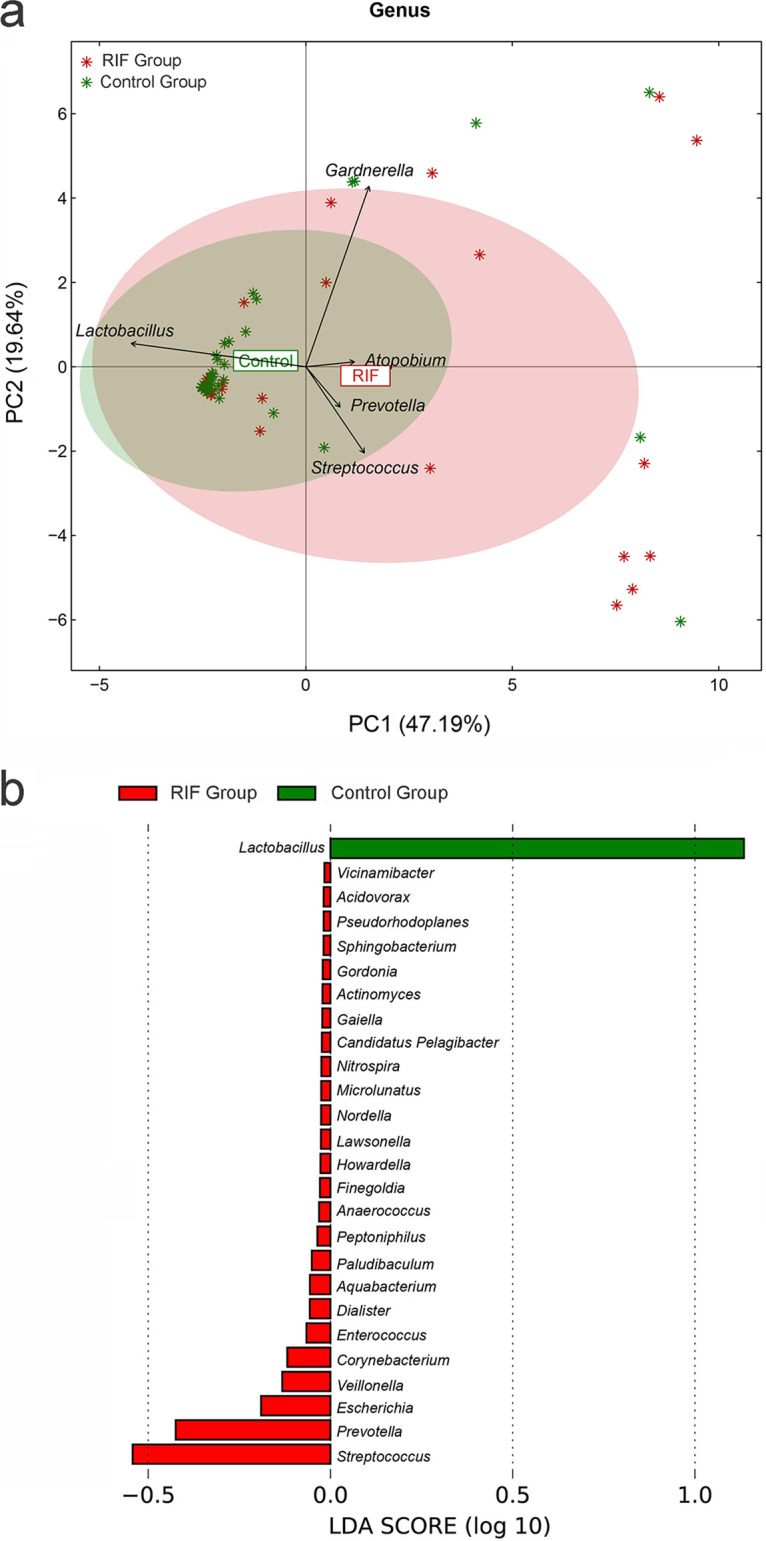
(a) A principal-component analysis was applied to demonstrate the distribution of the vaginal microbial communities in the samples. The arrows indicate the different genera, and their contributions to the explanation of the sample difference are shown by the arrow length. The angle between the arrows represents the positive correlation (<90°) or negative correlation (>90°) among the genera. (b) Linear discriminant analysis of the differentially abundant genera, which indicated their contribution to group differentiation. The green bar indicates that the genus (*Lactobacillus*) was more abundant in the control group, while red bars indicate that those genera were more abundant in the RIF group.

Principal-component analysis (PCA) was applied to illustrate the distribution of the microbial community in the samples (Fig. 3a). The spots belonging to the RIF group were dispersed, and half of them were scattered in the opposite direction of *Lactobacillus*, which is widely regarded as the probiotic that dominates the healthy female reproductive tract. Additionally, based on linear discriminant analysis (LDA), *Lactobacillus* was significantly decreased in the RIF group and contributed mostly to group differentiation (Fig. 3b). The dominant genus of both groups was *Lactobacillus*. The relative abundance of *Lactobacillus* was 85.766% ± 28.787% in the control group and significantly decreased (*P* value = 0.013) in the RIF group (61.833% ± 41.849%). Additionally, we defined the subjects for which *Lactobacillus* accounted for greater than 90% relative abundance as *Lactobacillus*-dominated (LD) samples and defined those for which *Lactobacillus* accounted for ≤90% as non-*Lactobacillus*-dominated (NLD) samples. The pregnancy rates of LD and NLD individuals were 72.723% and 34.723%, respectively, which presented a significant difference (*P* value = 0.006). This not only showed the link between vaginal *Lactobacillus* and pregnancy outcomes of the FET but also indicated that the vaginal microbial composition, especially the decrease in *Lactobacillus*, plays an important role in RIF pathogenesis.

### Comparative metabolomics in the vaginal environment.

Twenty-five samples were subjected to metabolic analysis, including 10 RIF group samples and 15 control samples (Table 2). Both the PCA results of the test and quality control (QC) samples and the correlation analyses of metabolites between the QC samples indicated the stability of the instrument and the good quality of the data (see Fig. S3). In this study, a total of 11,380 peaks of cations were obtained after noise removal; 449 nonredundant metabolites were annotated according to the mass-to-charge ratio (MS1), while 1,541 nonredundant metabolites were detected based on a molecular weight database (MS2). For the anions, 6,658 peaks were obtained, including 146 and 786 nonredundant metabolites according to MS1 and MS2, respectively. After removing the duplicated metabolites, we annotated 573 and 1,934 metabolites based on MS1 and MS2, respectively. As a result, we obtained 2,507 metabolites.

**TABLE 2:** Clinical characteristics of the participants in the two groups that were analyzed by metabolic analysis

Clinical characteristics	Value		*P* value
	RIF group (*n* = 10)	Control group (*n* = 15)	
Age (yrs)	33.7 ± 3.8	31.8 ± 3.8	0.189
BMI (kg/m^2^)	21.6 ± 3.5	20.9 ± 4.2	0.977
AMH (ng/ml)	3.8 ± 2.3	5.8 ± 4.7	0.338
Duration of infertility (yrs)	5.3 ± 3.4	3.9 ± 1.4	0.480
Endometrial thickness (mm)	10.2 ± 3.6	11.2 ± 2.1	0.382
No. of oocytes	13.5 ± 7.2	13.3 ± 6.7	0.955
No. of embryos	10.5 ± 7.2	10.5 ± 5.5	0.737
No. of high-quality embryos	2.2 ± 2.9	2.6 ± 2.3	0.498
No. of embryos transferred	2.0 ± 0.5	2.1 ± 0.5	0.658
No. of high-quality embryos transferred	1.3 ± 0.5	1.3 ± 0.9	0.628

10.1128/mBio.03242-19.3 FIG S3 Quality control results of the metabolomics analysis, including the PCA results of test and QC samples (a) and correlation analysis of metabolites between the QC samples (b). Download FIG S3, PDF file, 2.7 MB. Copyright © 2020 Fu et al. 2020 Fu et al. This content is distributed under the terms of the Creative Commons Attribution 4.0 International license.

**FIG 4:**
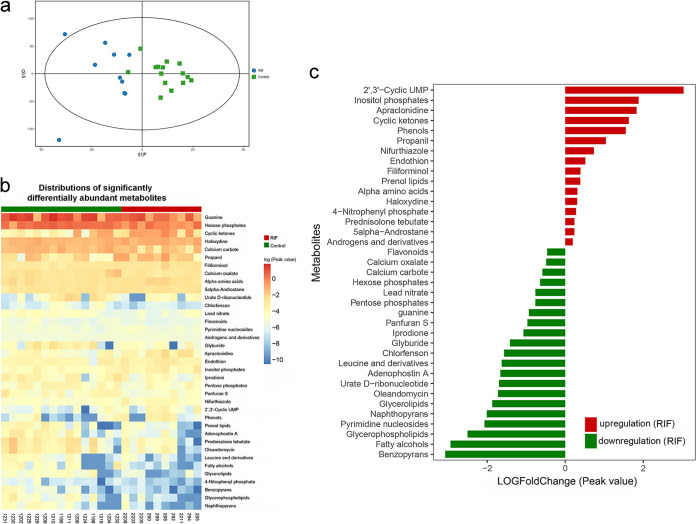
(a) Result of orthogonal projections to latent structures-discriminant analysis of the samples. The *x* axis shows the predicted principal-component score, which indicates the intergroup difference. The *y* axis represents the orthogonal principal-component score, which indicates the intragroup difference. (b) Heat map of the differentially abundant metabolites. (c) Quantitative fold change in the differentially abundant metabolites.

We utilized orthogonal projections to latent structures-discriminant analysis (OPLS-DA) to observe metabolites that were differentially abundant between these two groups and classify them into group-related and group-independent metabolites. The results indicated that the RIF group samples were clustered together and distinct from the grouped control samples (Fig. 4a). Of 2,507 annotated metabolites, 37 metabolites were found to have significant differences between the two groups, with variable importance for the projection (VIP) values of >1 and *P* values of <0.05 (Fig. 4b; Table S4). To quantify the up/downregulation of differentially abundant metabolites, we calculated the fold change in 37 differentially abundant metabolites. The results revealed that 16 metabolites were significantly upregulated in the RIF group, among which 2′,3-cyclic UMP and inositol phosphate were the top two metabolites, and they were upregulated by 4-fold or more; 21 metabolites were significantly downregulated in the RIF group, and 5 substances were downregulated by 4-fold or more: benzopyran, fatty alcohol, pyrimidine nucleoside, glycerophospholipid, and naphthopyran (Fig. 4c). The network of the metabolites that significantly correlated with each other was also investigated using Spearman’s correlation coefficients (*R* ≥ 0.6 or *R* ≤ −0.6) and *P* values (*P*＜0.05).

10.1128/mBio.03242-19.7 TABLE S4 Quantities of 37 metabolites that were found to have significant differences between the two groups via the determination of variable importance for the projection (VIP) values and *P* values. Download Table S4, DOCX file, 0.1 MB. Copyright © 2020 Fu et al. 2020 Fu et al. This content is distributed under the terms of the Creative Commons Attribution 4.0 International license.

### Correlation between the vaginal microbiota and metabolome.

**FIG 5:**
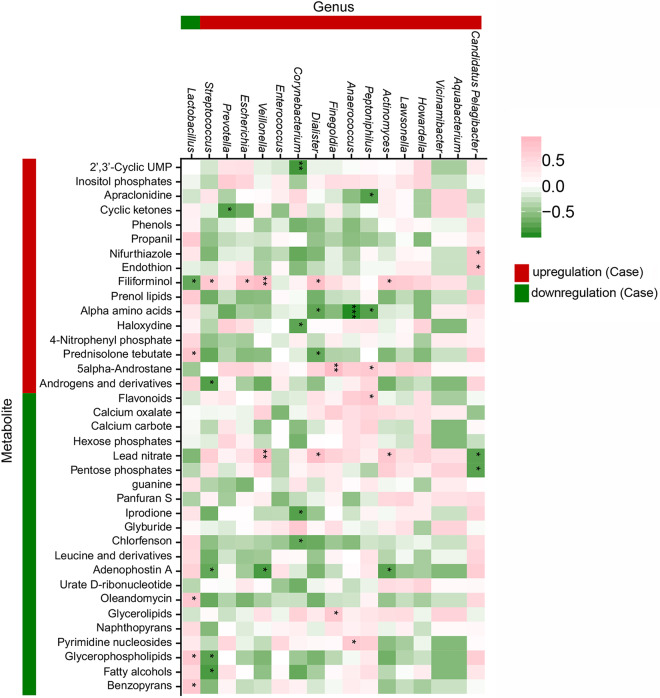
Correlation analysis between the differentially abundant metabolites and differential genera in the RIF group. Red on the bar outside the heat map indicates that the metabolite/genus was upregulated in the RIF group (case), while green indicates downregulation.

To further explore the pathogenic mechanisms of vaginal microbiota in RIF, we examined the correlation between the vaginal microbiota and metabolome. Although a one-to-one relationship between the metabolites and individual bacterial species could not be explained due to the lack of whole-genome information on the bacterial strains and the functional genes of microbial community based on the 16S rRNA sequencing method, the correlations in abundance between differential genera/species and metabolites were analyzed to explore the associated alteration in microbiota and metabolism in RIF patients. The correlations between the differentially abundant genera and metabolites were calculated in the RIF group (Fig. 5). The abundances of four metabolites (benzopyrans, glycerophospholipid, oleandomycin, and prednisolone tebutate) were significantly positively correlated with *Lactobacillus*, while filiforminol was negatively correlated with *Lactobacillus*. Among them, benzopyran and glycerophospholipids decreased more than 4-fold in the RIF group (*P* value < 0.05, *R* = 0.714), and this was associated with a significant reduction in *Lactobacillus* relative abundance in the RIF group. This result indicated that benzopyran and glycerophospholipids might function as metabolites during IVF, driven by *Lactobacillus* or related bacteria in the vaginal microbiota. Interestingly, *L. iners* was also positively correlated with benzopyran in the RIF group (*R* = 0.738). For the control group, consistent changes in benzopyrans with the relative abundance of *L. iners* were identified (*R* = 0.527).

## DISCUSSION

This study is an attempt to explain the influence of the vaginal microbiota and metabolites on RIF patients while using first-cycle successful FET cases as the control. The vaginal microbiota structure of the RIF group was different from that of the control group, containing significantly decreased *Lactobacillus* and harboring more potential pathogens. The metabolite profile delineated 37 metabolites to be significantly differentially abundant between the two groups, and this may have resulted in or from the changes in the microbiota composition.

Considering that the hormone level, menstrual cycle, cause of infertility, and pregnancy status were closely associated with the female vaginal microbiota and metabolites, these factors were all homogenized in this study by sampling consistently on the same day of embryo transfer in the FET cycle, and only patients with unexplained RIF without any other diseases who were confirmed to not be pregnant in this cycle were recruited. The baseline parameters (age, body mass index [BMI], anti-Müllerian hormone [AMH], duration of infertility, and endometrial thickness) and the number of embryos showed no significant differences in the two groups (Table 1). Notably, although the number of embryos transferred was significantly lower in the RIF group, it should not cause a confounding bias for the results, as it was determined by the clinical treatment characteristics of the RIF patients. To achieve a higher implantation and live-birth rate, doctors usually transfer 1 to 2 blastocysts for RIF patients while transferring 2 to 3 cleavage-stage embryos for the patients in the first FET cycle to avoid cycle cancellation due to blastocyst culture (37–39).

The abundances of 26 genera detected in the present study were significantly different between the two groups (*P* value < 0.05). In the RIF group, all of the genera were significantly increased, especially the aerobic bacteria (8.593% for the RIF group and 2.349% for the control group, *P* value < 0.05), whereas only *Lactobacillus* abundance was significantly reduced. Among the 25 increased genera, many anaerobic bacteria, such as *Gardnerella*, *Prevotella*, and *Atopobium*, are the main pathogens for bacterial vaginosis (BV), while the abundance of many aerobic bacteria, such as *Escherichia*, *Enterococcus*, *Streptococcus*, and *Corynebacterium*, are closely related to the risk of aerobic vaginitis (AV) and urinary tract infection (UTI). A study of asymptomatic reproductive tract infections in couples undergoing IVF showed that 29.1% (83/285) of the males and 26.3% (75/285) of the females carried at least one potential pathogen for reproductive tract disease, and more positive cases were found in the IVF pregnancy failure group than in the IVF control group. Additionally, Enterococcus faecalis, Escherichia coli, Streptococcus agalactiae, and Gardnerella vaginalis were significantly associated with decreased levels of *Lactobacillus* (*P* value < 0.01) (40). Similarly, the abundance of *Lactobacillus* was negatively related to the abundance of potential BV/AV pathogens in this study, including *Gardnerella*, *Prevotella*, *Atopobium*, and *Streptococcus*, which indicated the protective function of *Lactobacillus*. Our results also indicated that *Lactobacillus* was losing its dominant position in the RIF group, which may be associated with the occurrence of repeated pregnancy failures. Furthermore, the pregnancy rate of NLD subjects was significantly decreased compared with that of LD subjects. Although the relative abundance of *Lactobacillus* was significantly lower in the RIF group than in the control group, the LDA value was still too low to be identified as a biomarker to predict the occurrence of RIF. However, the ROC curve showed that when *Veillonella* was factored in, the sensitivity and specificity both increased, demonstrating that the combination of *Lactobacillus* and *Veillonella* can be used as a potential biomarker for the prediction of RIF occurrence.

An increase in vaginal microbial diversity in RIF patients was observed in this study, which is consistent with the study by Hyman et al. in 2012 (35). That report was the first study to suggest that the species diversity index of the vaginal microbiota distinguished women who had live births from those who did not (35). Another study also showed that the clinical pregnancy rate and live-birth rate were significantly reduced (*P* value = 0.01) in a cohort with a vaginal Shannon-Wiener index higher than 0.93 (41). To date, few vaginal microbiota studies have investigated RIF patients, but there are association studies of an abnormal vaginal microbiota with IVF. A review concluded that culture-dependent studies indicated that an abnormal vaginal microbiota was not linked to the outcome of IVF. However, this conclusion was overturned by high-throughput sequencing results, which elaborated that microbiota disorders negatively influenced the IVF outcome (42). Another review demonstrated that the incidence of bacterial vaginosis (BV) was associated with early abortion of IVF pregnancies and infertility due to tubal factors and that it was not associated with the pregnancy rate and live-birth rate (30).

To evaluate the difference in the vaginal metabolites between the RIF and control groups, we identified the fold changes of 37 significantly different metabolites in two groups. In the RIF group, 2′,3-cyclic UMP and inositol phosphate increased by 4-fold or more, while benzopyran, fatty alcohol, pyrimidine nucleoside, glycerophospholipid, and naphthopyran decreased by 4-fold or more. As inositol phosphate is one of the metabolites of glycerophospholipid (43), the significant increase in inositol phosphate should be linked to the decrease in glycerophospholipid, which may result in the reduction of other important metabolites from glycerophospholipid involved in the embryo implantation process due to the lack of substances, such as lysobisphosphatidic acid and prostaglandin (44–49). In addition, the increase in inositol phosphate could promote the internal influx of Ca^2+^ and lead to contraction of the uterine, which is detrimental to embryo implantation (50). Benzopyran and naphthopyran are selective estrogen receptor modulators that can both influence the outcome of IVF. Interestingly, after analyzing the association between significantly different metabolites and genera, benzopyran and glycerophospholipid were listed in the 4 metabolites that were significantly positively correlated with the abundance of the key vaginal genus *Lactobacillus*. Thus, these two metabolites are further discussed.

Glycerophospholipids are important structural and regulatory components of biofilms and serve as precursors for many active biomolecules, such as arachidonic acid (AA) and lysobisphosphatidic acid (LPA), which are catalyzed by phospholipase A_2_ (PLA_2_) (45, 49). AA then produces prostaglandin (PG) under the action of cyclooxygenase 2 (COX-2). PG and LPA are the terminal products of glycerophospholipids that play key roles in embryo implantation. According to a previous study, LPA plays an essential role in maintaining the normal size and spacing of the embryo, which is positively correlated with embryo implantation in mice (44). LPA_3_, a G protein-coupled receptor that functions in the uterine epithelium, also regulates the activity of COX-2 and the level of PGs, which could directly affect implantation and decidualization (46, 48). Mice lacking LPA_3_ showed defects such as deferred on-time implantation, delayed fetal development, embryo crowding, and sharing of one placenta by several embryos. Additionally, deviation in the PG-producing pathway has a significant impact on the implantation process, resulting in a reduction in the likelihood of achieving pregnancy (49). Furthermore, it has been reported that levels of endometrial LPA_3_ were reduced in RIF patients (49) and that its activation induces decidualization (48). More importantly, phospholipase could be stimulated by lipopolysaccharide, which is the key component of the outer membrane of Gram-negative bacteria, such as *Lactobacillus*. In addition, glycerophospholipids were positively correlated with the abundance of *Lactobacillus*. Hence, a balanced vaginal microbiota would result in the normal regulation of glycerophospholipids, which might lead to successful embryo implantation.

Sex hormones are potential metabolites functioning during IVF. In this study, androgens, 5α-androstane, and other androgen derivatives were all upregulated in the RIF group, which may antagonize estrogen secretion, inhibit endometrial hyperplasia, depress ovarian and pituitary function, and, in turn, cause a negative effect on embryo implantation. Benzopyran, flavonoid, and naphthopyran, as selective estrogen receptor modulators (SERMs) (51), were all downregulated in the RIF group. Among them, benzopyran was downregulated more than 4-fold. SERMs such as clomiphene and letrozole, which are commonly used to promote follicular development, bind with high affinity to the estrogen receptor to induce agonistic or antagonistic activities in special tissues (52). Moreover, benzopyran has been identified as a secondary metabolite not only in green plants but also in bacteria and fungi, and it has been routinely employed as an herbal remedy for decades (53). Benzopyran is currently used as an anticoagulant because it is a COX-2-inhibitor (54), but its effect on the reproductive system has not been studied. However, aspirin has been known as an irreversible inhibitor of COX, and its anticoagulant effect plays a significant role in the treatment of recurrent spontaneous abortion (55). Hence, benzopyran may also play a similar role in embryo implantation as aspirin. Furthermore, although the quantity of benzopyran was positively correlated with the abundance of *L. iners* in our study, the relationship between benzopyran and vaginal microbiota and its specific functions in embryo implantation still need further investigation.

Our discovery of a significant difference in the vaginal microbiota and metabolomes of RIF patients establishes an important theoretical grounding to the potential development of the microbiota and metabolome as biomarkers for predicting RIF occurrence. Although previous studies have demonstrated that the endometrial microbiota is associated with infertility and IVF (56–58), vaginal rather than endometrial samples are more feasible for clinical application due to the lower contamination risk and noninvasive sample process. First, unlike vaginal specimen sampling, endometrial samples are easily contaminated by the cervicovaginal microbiome due to the transvaginal collection and the 4-fold fewer bacteria inhabiting the endometrium than the vagina (37). Second, endometrial biopsy specimens are usually not taken in the WOI of the same cycle because of endometrial damage caused by the invasive procedure; hence, the result from the endometrial biopsy specimen cannot show the condition of the patient at the time of embryo transfer. Moreover, other than endometrial biopsy specimens, endometrial fluid and uterine flushing fluid are the other endometrial samples commonly used in the clinic. As the microecology shows inconsistent characteristics in these three environments, it is hard to distinguish which is more persuasive (59). Nevertheless, despite the significantly different genera and metabolites in RIF patients’ vaginal environments in this study, further verification is still needed. Furthermore, there are limitations of the 16S rRNA sequencing approach, such as the short-read lengths obtained, sequencing errors (60, 61), differences arising from the different regions chosen (62), and difficulties in assessing operational taxonomic units (OTUs) (63). Also, limited microbial taxonomic messages beyond the bacteria will be obtained from 16S rRNA sequences because of the low coverage of chlamydia, archaeal, fungal, chloroplastic, mitochondrial, and eukaryotic rRNA genes (64). Hence, additional biomarkers should be detected but should not be based solely on the taxonomic information from the 16S rRNA sequencing approach. Functional genes should also be complemented and associated with metabolites for microbial metabolic pathway network construction to explore RIF pathogenesis.

### Conclusion.

The vaginal microbiota of RIF patients presented higher microbial diversity and lower abundance of *Lactobacillus*, which significantly associated with the pregnancy rate. LPA and PG metabolized from glycerophospholipid are key factors affecting implantation and decidualization, and benzopyran may contribute to the outcome of pregnancy as a SERM.

## MATERIALS AND METHODS

### Criteria for recruitment of the research subjects and ethical approval.

Patients who underwent frozen embryo transfer (FET) in the Department of Reproductive Medicine, Peking University Shenzhen Hospital, from May 2018 to December 2018 were selected and divided into the unexplained RIF group (RIF group) and successful pregnancy in the first FET cycle group (control group). All of the women were less than 40 years of age, and their functional ovarian reserve was assessed by levels of follicle stimulation hormone (FSH) of <12 mIU/ml and AMH of >1.1 ng/ml. The inclusion criteria for the RIF group were patients who (i) received at least four good-quality embryos in a minimum of three fresh or frozen cycles but had still not become pregnant and (ii) were not pregnant in this FET cycle. The patients who were counted in the control group were those undergoing an embryo transfer for the first time, and this cycle was FET. In addition, these patients were confirmed to be pregnant in this FET cycle.

Patients with simple male factor infertility, ovulation and menstrual disorders, genital tract organic lesions, and systemic diseases were excluded. All patients were confirmed to have a normal uterine cavity by hysteroscopy and a normal chromosome karyotype by chromosome G-banding and karyotype analysis. The patients all underwent examinations of the cervical mucus and vaginal secretions at 1 month and 7 days before embryo transfer. Patients showing bacterial vaginosis, vulvovaginal candidiasis, trichomonas vaginitis, Chlamydia trachomatis, Ureaplasma urealyticum, or Neisseria gonorrhoeae infection, or other vaginal subjective symptoms, such as vaginal itching and abnormal discharge, at the examination at either 1 month or 7 days before transfer were excluded. Patients receiving any antibiotics (oral or topical) or vaginal douching or engaging in sexual behavior within 2 weeks before sample collection were also excluded.

The study was approved by the Ethics Committee of Peking University Shenzhen Hospital on 10 May 2018. The study is sponsored by the Peking University Shenzhen Hospital (2018 no. 017) and was conducted as a single-center study without any investigational product. All enrolled subjects provided written consent and gave permission for access to medical records to obtain their related clinical information and vaginal specimens.

### Sample processing.

Vaginal specimens were collected on the day of embryo transfer before the operation. A sterile sample collection kit (CY-90003T, iCleanhcy; Huachenyang [Shenzhen] Technology Co., Ltd., China) with a separated package was used in the sample collection, and it comprised a long-handle swab connected to a sealed cap and inserted in a collection tube. Each sample kit was weighed before sample collection as the dry weight. During the sampling, the swab was carefully taken out of the tube and immersed into the upper third of the vagina and maneuvered in a circle by holding the cap constantly to avoid contamination of the swab. After sampling, the swab with vaginal discharge was placed back into the tube and weighed as the wet weight. The net weight of the vaginal discharge was recorded as the wet weight minus the dry weight. Two parallel samples were taken from each patient, immediately placed in an ice box, and then transferred to a −80°C freezer within 20 min for subsequent 16S rRNA gene sequencing and metabolic analysis (Shenzhen We-Health Gene Company).

### Total DNA extraction and 16S rRNA sequencing.

DNA extraction was conducted with a DNeasy PowerSoil kit (Qiagen, Hilden, Germany) followed by DNA purification with VAHTS DNA Clean Beads (Vazyme, Nanjing, China) according to the manufacturers’ instructions. The V3-V4 region of the 16S rRNA gene was amplified by PCR with the universal primers 338F (5′-ACTCCTACGGGAGGCAGCAG-3′) and 806R (5′-GGACTACHVGGGTWTCTAAT-3′) using a TransStart FastPfu DNA polymerase kit on an ABI GeneAmp 9700 PCR instrument. The PCR system was 50 μl in total, and it contained 4 μl of 5× FastPfu buffer, 2 μl of 2.5 mM deoxynucleoside triphosphates (dNTPs), 0.8 μl of forward primer, 0.8 μl of reverse primer, 0.4 μl of FastPfu Polymerase, 0.2 μl of bovine serum albumin (BSA), 10 ng of template DNA, and double-distilled water (ddH_2_O) to make up the total volume. PCR was performed using the following conditions: 3 min of denaturation at 95°C, 29 cycles of denaturation at 95°C for 30 s, annealing at 45°C for 30 s, and elongation at 72°C for 45 s, and a final extension at 72°C for 10 min. The 16S rRNA gene amplicons were purified and used for MiSeq sequencing library construction according to the manufacturer’s instructions for the MiSeq reagent kit v2.

### Microbiota analysis.

Clean paired-end sequences were filtered by eliminating low-quality base pairs, contaminated reads, N-containing reads, and low-complexity sequences. The operational taxonomic units (OTUs) were clustered under the 97% cutoff and annotated by the Ribosomal Database Project after merging reads via overlaps. The α-diversity inferred by the Shannon-Wiener index was calculated by the R package vegan; meanwhile, a principal-component analysis (PCA) was performed by the ade4 package. Linear discriminant analysis (LDA) was performed using Galaxy software. A random forest model was constructed between the RIF group and control group with 5-fold cross-validation using the R package randomForest (65). The genera selected by Gini coefficient were used for a receiver operating characteristic (ROC) analysis, and the area under the curve (AUC) was calculated.

### Metabolome analysis.

Metabolites were extracted from swabs by adding 20 μl isolation liquid (methanol/acetonitrile/water [2:2:1]) to each 1 mg vaginal discharge. Then, 10 μl of isolates from all samples were mixed as quality control (QC) samples for stability evaluation during the experiment (66, 67), 2 μl of isolates of each sample was analyzed by liquid chromatography (LC) and mass spectrometry (MS) (Q-Exactive Orbitrap; Thermo-Fisher Scientific, USA), and the signals of metabolites in all samples were examined. Using ProteoWizard (v3.0.9134) and the XCMS package in R (v3.2), the MS raw data were converted to a data matrix that contained the retention time (RT), mass-to-charge ratio (*m/z*) value, and peak intensity. All output data were normalized by the internal standard normalization method and are presented as the peak value (peak area of the test sample/peak area of internal standard sample). According to the peak values of metabolites, their variable importance in the projection (VIP) was calculated by orthogonal projections to latent structures-discriminant analysis (OPLS-DA). Student's *t* test was applied to detect differentially enriched metabolites between the control and RIF groups (VIP > 1, *P* value < 0.05). Correlations among the metabolites were evaluated by Spearman’s correlation analysis, and the network was visualized by Cytoscape software (v3.4.0). In addition, a Spearman correlation analysis was also applied to investigate the relationship between the vaginal microbiota and metabolites.

### Data availability.

The 16S rRNA gene sequencing data for the 67 vaginal microbiota samples analyzed in this study have been deposited with the National Center for Biotechnology Information (NCBI) under reference number PRJNA590580. The metabolome profiles of the final identified 2,507 metabolites are shown in Table S5 in the supplemental matieral.

10.1128/mBio.03242-19.8 TABLE S5 Metabolome profile of the final identified 2,507 metabolites. Download Table S5, XLSX file, 0.1 MB. Copyright © 2020 Fu et al. 2020 Fu et al. This content is distributed under the terms of the Creative Commons Attribution 4.0 International license.
