# Supplementary figures and images for: Alterations in Vaginal Microbiota and Associated Metabolome in Women with Recurrent Implantation Failure
Source: mBio. 2020 Jun 2;11(3):e03242-19. doi: 10.1128/mBio.03242-19 (PMC7267891; doi:10.1128/mBio.03242-19)

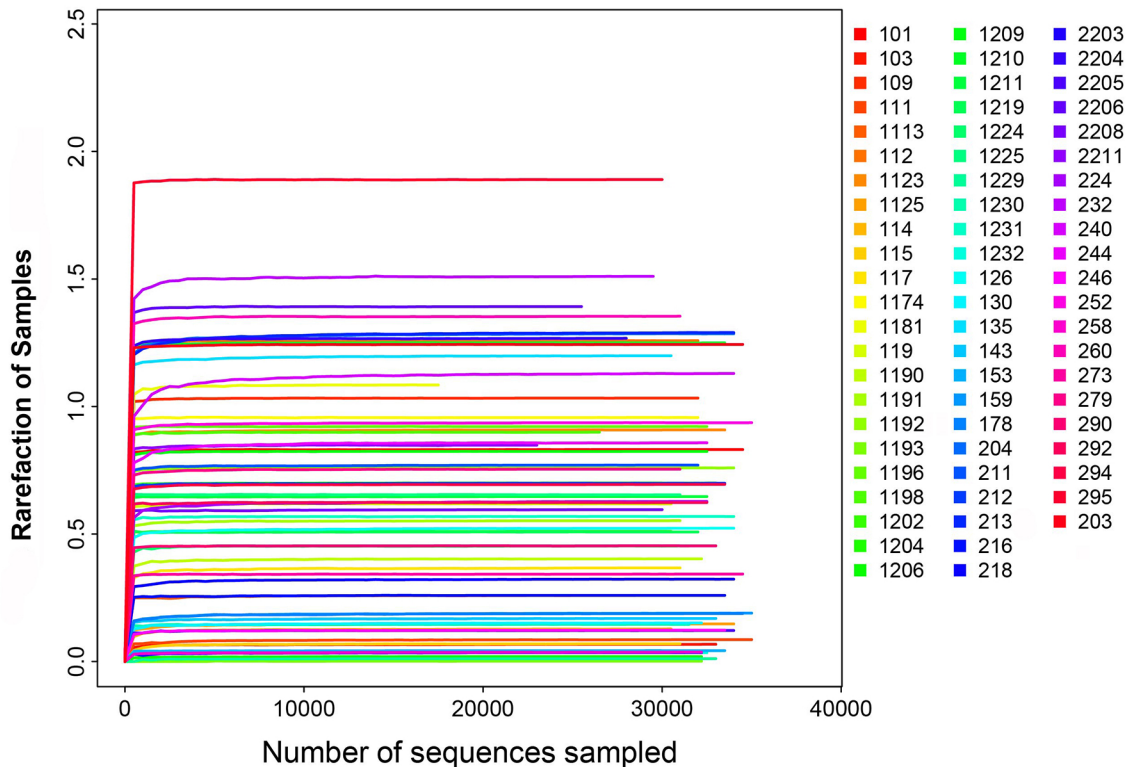

Supplement: FIG S1 [file mBio.03242-19-sf001.pdf]

# ROC Curve

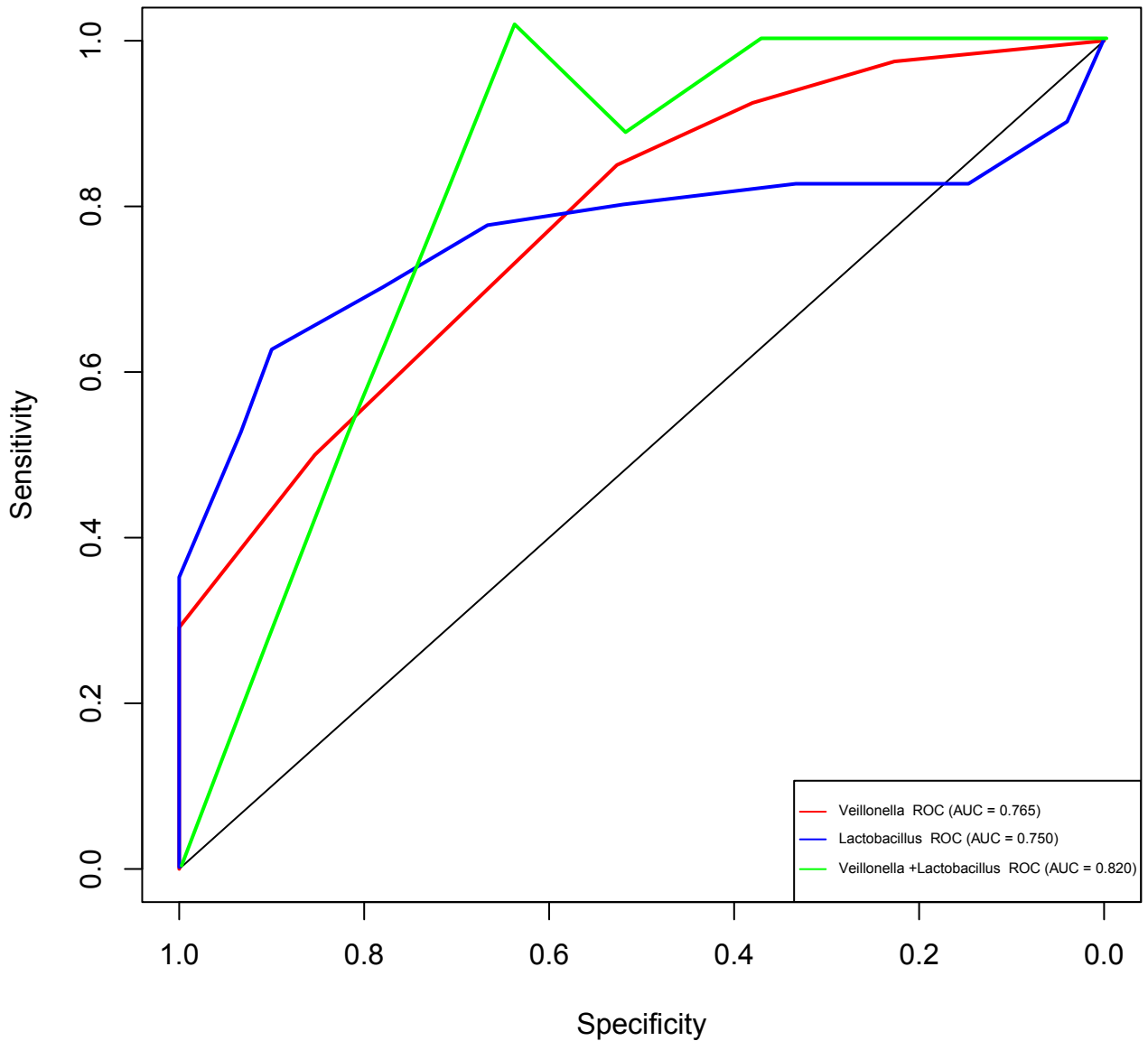

Supplement: FIG S2 [file mBio.03242-19-sf002.pdf]

a

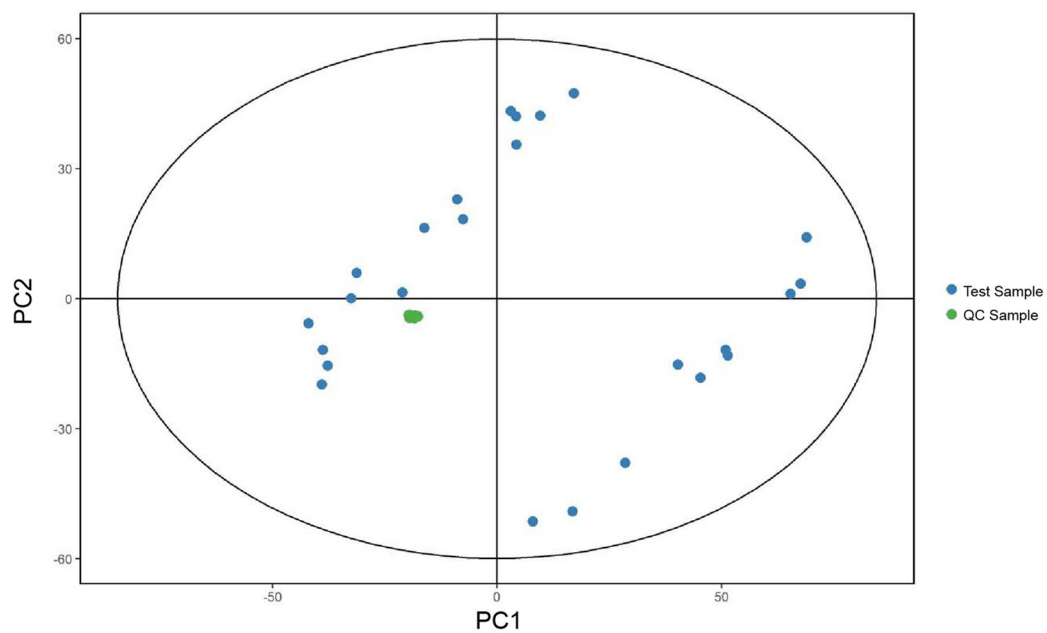

b

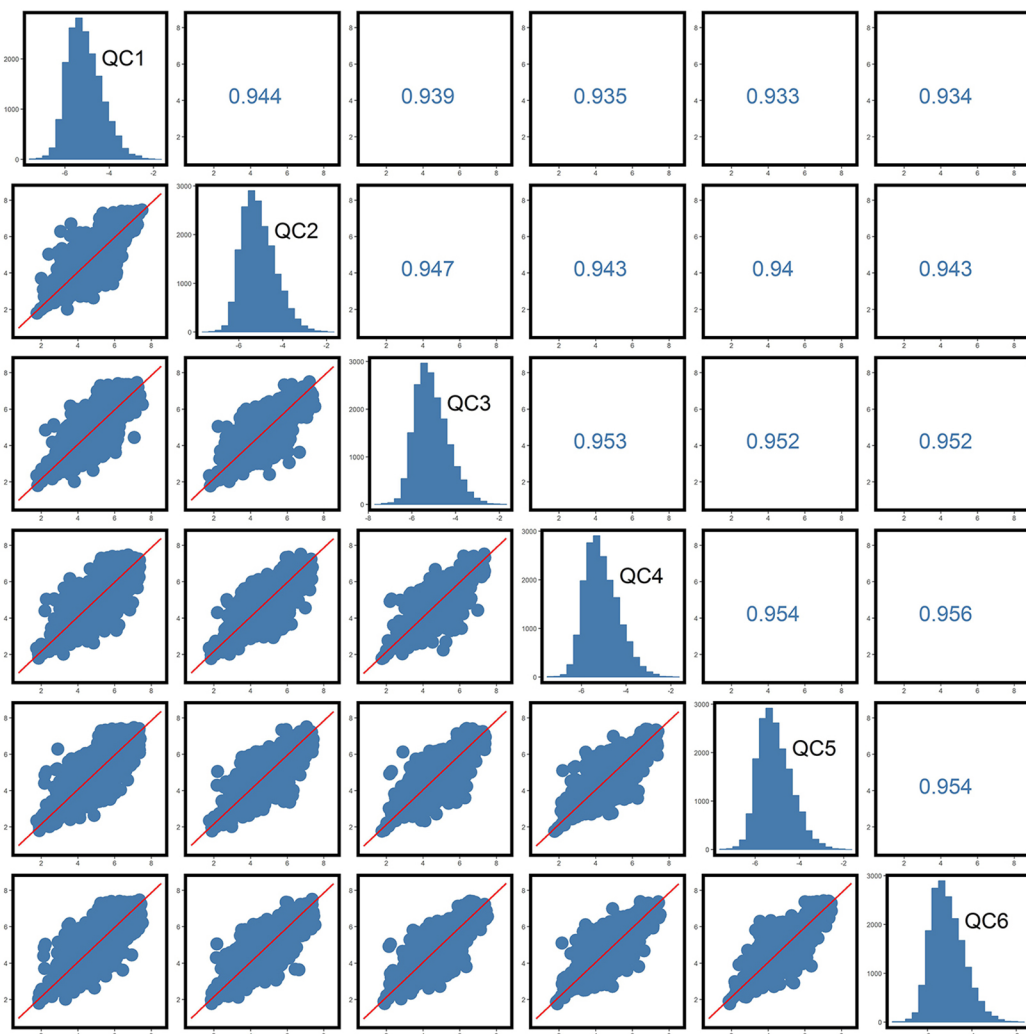

Supplement: FIG S3 [file mBio.03242-19-sf003.pdf]
