# Supplementary material for: Alterations in Vaginal Microbiota and Associated Metabolome in Women with Recurrent Implantation Failure
Source: mBio. 2020 Jun 2;11(3):e03242-19. doi: 10.1128/mBio.03242-19 (PMC7267891; doi:10.1128/mBio.03242-19)
Supplement: TABLE S1 [file mBio.03242-19-st001.docx]

**Supplementary Table 1.** Genera in the vaginal microbiota that were significantly differentially abundant between the RIF and control groups.

| Genus | RIF group（n=27）  Relative abundance (%) | Control group（n=40）  Relative abundance (%) | *P*-value |
| --- | --- | --- | --- |
| *Lactobacillus* | 61.833±41.849 | 85.766±28.787 | 0.013 |
| ^#^ *Streptococcus* | 6.457±19.815 | 1.974±12.177 | 0.022 |
| *Prevotella* | 3.58±10.221 | 1.078±4.787 | 0.027 |
| ^#^ *Enterococcus* | 0.335±1.292 | 0.256±1.591 | 0.015 |
| ^#^*Dialister* | 0.279±1.275 | 0.069±0.346 | 0.015 |
| *Finegoldia* | 0.19±0.248 | 0.069±0.204 | 0.030 |
| ^#^*Corynebacterium* | 0.52±2.268 | 0.04±0.118 | 0.009 |
| *Anaerococcu* | 0.136±0.296 | 0.034±0.083 | 0.023 |
| *Veillonella* | 0.998±5.058 | 0.019±0.103 | 0.000 |
| *Peptoniphilu* | 0.076±0.121 | 0.016±0.028 | 0.018 |
| ^#^*Escherichia* | 1.012±3.408 | 0.01±0.034 | 0.033 |
| *Actinomyces* | 0.046±0.142 | 0.002±0.006 | 0.005 |
| *Acidovorax* | 0.016±0.054 | 0.001±0.004 | 0.024 |
| *Lawsonella* | 0.025±0.051 | 0.004±0.007 | 0.008 |
| *Howardella* | 0.021±0.071 | 0.001±0.003 | 0.035 |
| *Sphingobacterium* | 0.006±0.018 | 0±0.003 | 0.026 |
| *Vicinamibacter* | 0.008±0.025 | 0±0.001 | 0.005 |
| *Aquabacterium* | 0.003±0.005 | 0±0.001 | 0.012 |
| *Nitrospira* | 0.002±0.005 | 0±0 | 0.022 |
| *Nordella* | 0.022±0.076 | 0±0 | 0.005 |
| *Gaiella* | 0.005±0.013 | 0±0 | 0.013 |
| *Paludibaculum* | 0.002±0.007 | 0±0 | 0.034 |
| *Pseudorhodoplanes* | 0.001±0.005 | 0±0 | 0.034 |
| *Gordonia* | 0.001±0.003 | 0±0 | 0.034 |
| *Candidatus* | 0.001±0.002 | 0±0 | 0.034 |
| *Microlunatus* | 0.001±0.002 | 0±0 | 0.034 |

^#^ Aerobe or facultative aerobes
