# Supplementary material for: Alterations in Vaginal Microbiota and Associated Metabolome in Women with Recurrent Implantation Failure
Source: mBio. 2020 Jun 2;11(3):e03242-19. doi: 10.1128/mBio.03242-19 (PMC7267891; doi:10.1128/mBio.03242-19)
Supplement: TABLE S2 [file mBio.03242-19-st002.docx]

**Supplementary Table 2.** Relative abundance of *Lactobacillus* species discovered in the samples that were significantly different between the RIF and control groups.

| *Lactobacillus* species | RIF group（n=27）  Relative abundance (%) | Control group（n=40）  Relative abundance (%) | *P*-value |
| --- | --- | --- | --- |
| *Lactobacillus iners* | 23.493±39.266 | 40.402±42.806 | 0.028* |
| *Lactobacillus crispatus* | 33.212±41.360 | 30.820±38.102 | 0.606 |
| *Lactobacillus jensenii* | 4.421±11.958 | 12.659±23.660 | 0.369 |
| *Lactobacillus paracasei* | 1.007±5.030 | 0.000±0.001 | 0.863 |
| *Lactobacillus reuteri* | 0.151±0.358 | 0.496±1.639 | 0.594 |
| *Lactobacillus gasseri* | 0.068±0.216 | 0.060±0.234 | 0.263 |
| *Lactobacillus salivarius* | 0.000±0.001 | 0.031±0.196 | 0.884 |
| *Lactobacillus coleohominis* | 0.000±0.000 | 0.025±0.096 | 0.114 |
| *Lactobacillus intestinalis* | 0.003±0.007 | 0.002±0.003 | 0.816 |
| *Lactobacillus fermentum* | 0.002±0.008 | 0.001±0.003 | 0.305 |
| *Lactobacillus mucosae* | 0.000±0.000 | 0.001±0.004 | 0.453 |
| *Lactobacillus acetotolerans* | 0.000±0.000 | 0.001±0.003 | 0.453 |

^*^ *P*-value < 0.05，statistical significantly different
