# Supplementary material for: Alterations in Vaginal Microbiota and Associated Metabolome in Women with Recurrent Implantation Failure
Source: mBio. 2020 Jun 2;11(3):e03242-19. doi: 10.1128/mBio.03242-19 (PMC7267891; doi:10.1128/mBio.03242-19)
Supplement: TABLE S3 [file mBio.03242-19-st003.docx]

| Genus | Gini coefficient | Genus | Gini coefficient |
| --- | --- | --- | --- |
| *Veillonella* | 2.219860501 | *Dialister* | 1.413890801 |
| *Lactobacillus* | 2.184697004 | *Anaerococcus* | 1.175928109 |
| *Prevotella* | 1.701806024 | *Actinomyces* | 1.166650653 |
| *Acinetobacter* | 1.678297733 | *Gardnerella* | 1.158224737 |
| *Lawsonella* | 1.615692114 | *Staphylococcus* | 1.116343361 |
| *Streptococcus* | 1.561401876 | *Curvibacter* | 1.082722866 |
| *Finegoldia* | 1.557360308 | *Escherichia* | 1.05947554 |
| *Corynebacterium* | 1.555693029 | *Pseudomonas* | 0.799294279 |
| *Peptoniphilus* | 1.440680637 | *Ralstonia* | 0.626080223 |

**Supplementary Table 3.** Gini coefficient calculated by Random Forest algorithm among 18 genera whose abundance were both above 0 in two groups out of 26 significantly different genera.
