# Supplementary material for: Alterations in Vaginal Microbiota and Associated Metabolome in Women with Recurrent Implantation Failure
Source: mBio. 2020 Jun 2;11(3):e03242-19. doi: 10.1128/mBio.03242-19 (PMC7267891; doi:10.1128/mBio.03242-19)
Supplement: TABLE S4 [file mBio.03242-19-st004.docx]

**Supplementary Table 4.** Quantities of 37 metabolites that were found to have significant differences between the two groups via the determination of variable importance for the projection (VIP) values and *P*-values.

| Metabolites | RIF group (n=10)  Peak value | | Control group (n=15)  Peak value | | VIP value | | *P*-value | |
| --- | --- | --- | --- | --- | --- | --- | --- | --- |
| Guanine | | 1.409±1.000 | | 2.687±1.614 | 2.688 | 0.036 | |  |
| 5alpha-Androstane | | 0.077±0.013 | | 0.066±0.013 | 2.311 | 0.041 | |  |
| Androgens and derivatives | | 0.006±0.001 | | 0.005±0.001 | 2.540 | 0.023 | |  |
| Endothion | | 0.035±0.014 | | 0.025±0.010 | 2.740 | 0.033 | |  |
| Nifurthiazole | | 0.017±0.009 | | 0.010±0.002 | 2.860 | 0.044 | |  |
| Calcium oxalate | | 0.062±0.010 | | 0.087±0.040 | 1.384 | 0.033 | |  |
| Flavonoids | | 0.004±0.001 | | 0.005±0.002 | 1.466 | 0.043 | |  |
| Lead nitrate | | 0.006±0.004 | | 0.011±0.005 | 3.420 | 0.025 | |  |
| Panfuran S | | 0.114±0.028 | | 0.150±0.045 | 2.980 | 0.034 | |  |
| Pyrimidine nucleosides | | 0.003±0.002 | | 0.011±0.015 | 2.547 | 0.045 | |  |
| Chlorfenson | | 0.002±0.001 | | 0.005±0.005 | 1.836 | 0.030 | |  |
| 4-Nitrophenyl phosphate | | 0.001±0.001 | | 0.002±0.003 | 2.983 | 0.048 | |  |
| Prednisolone tebutate | | 0.012±0.011 | | 0.052±0.053 | 3.461 | 0.012 | |  |
| Benzopyrans | | 0.001±0.001 | | 0.005±0.007 | 3.428 | 0.017 | |  |
| Prenol lipids | | 0.006±0.006 | | 0.015±0.014 | 2.112 | 0.038 | |  |
| Calcium carbote | | 0.161±0.100 | | 0.241±0.068 | 1.605 | 0.025 | |  |
| Pentose phosphates | | 0.012±0.009 | | 0.021±0.010 | 1.562 | 0.038 | |  |
| Iprodione | | 0.014±0.010 | | 0.028±0.021 | 1.105 | 0.029 | |  |
| 2',3'-Cyclic UMP | | 0.017±0.015 | | 0.006±0.006 | 2.540 | 0.046 | |  |
| Alpha amino acids | | 0.094±0.024 | | 0.076±0.020 | 2.423 | 0.045 | |  |
| Apraclonidine | | 0.050±0.039 | | 0.014±0.010 | 3.649 | 0.017 | |  |
| Glyburide | | 0.012±0.009 | | 0.032±0.031 | 1.902 | 0.027 | |  |
| Propanil | | 0.211±0.145 | | 0.103±0.112 | 2.181 | 0.046 | |  |
| Haloxydine | | 0.316±0.071 | | 0.256±0.038 | 2.490 | 0.027 | |  |
| Filiforminol | | 0.071±0.022 | | 0.054±0.012 | 2.159 | 0.042 | |  |
| Hexose phosphates | | 0.905±0.463 | | 1.412±0.369 | 1.634 | 0.006 | |  |
| Adenophostin A | | 0.006±0.009 | | 0.020±0.019 | 1.769 | 0.024 | |  |
| Urate D-ribonucleotide | | 0.011±0.010 | | 0.037±0.045 | 1.488 | 0.050 | |  |
| Phenols | | 0.020±0.020 | | 0.007±0.011 | 1.058 | 0.045 | |  |
| Oleandomycin | | 0.013±0.013 | | 0.043±0.046 | 2.241 | 0.027 | |  |
| Glycerophospholipids | | 0.002±0.003 | | 0.011±0.013 | 2.414 | 0.018 | |  |
| Fatty alcohols | | 0.002±0.002 | | 0.012±0.014 | 1.317 | 0.012 | |  |
| Naphthopyrans | | 0.002±0.03 | | 0.008±0.012 | 2.273 | 0.033 | |  |
| Inositol phosphates | | 0.032±0.018 | | 0.017±0.007 | 2.361 | 0.028 | |  |
| Glycerolipids | | 0.002±0.003 | | 0.009±0.007 | 1.843 | 0.004 | |  |
| Leucine and derivatives | | 0.003±0.004 | | 0.009±0.010 | 1.364 | 0.033 | |  |
| Cyclic ketones | | 0.357±0.236 | | 0.115±0.160 | 1.358 | 0.006 | |  |
